# Toward a Robust Multi-Objective Metaheuristic for Solving the Relay Node Placement Problem in Wireless Sensor Networks

**DOI:** 10.3390/s19030677

**Published:** 2019-02-07

**Authors:** José M. Lanza-Gutiérrez, Nuria Caballé, Juan A. Gómez-Pulido, Broderick Crawford, Ricardo Soto

**Affiliations:** 1 Escuela Técnica Superior de Ingenieros Industriales, Centro de Electrónica Industrial, Universidad Politécnica de Madrid, 28006 Madrid, Spain; 2 Facultad de Farmacia, Campus Montepríncipe, Universidad CEU San Pablo, 28668 Madrid, Spain; nuria.caballecervigon@ceu.es; 3 Escuela Polítécnica, Universidad de Extremadura, 10003 Cáceres, Spain; jangomez@unex.es; 4 Escuela de Ingeniería Informática, Pontificia Universidad Católica de Valparaíso, 2362807 Valparaíso, Chile; broderick.crawford@pucv.cl (B.C.); ricardo.soto@pucv.cl (R.S.)

**Keywords:** deployment, energy cost, metaheuristic, multi-objective, relay node, reliability, sensitivity, wireless sensor network

## Abstract

During the last decade, Wireless sensor networks (WSNs) have attracted interest due to the excellent monitoring capabilities offered. However, WSNs present shortcomings, such as energy cost and reliability, which hinder real-world applications. As a solution, Relay Node (RN) deployment strategies could help to improve WSNs. This fact is known as the Relay Node Placement Problem (RNPP), which is an NP-hard optimization problem. This paper proposes to address two Multi-Objective (MO) formulations of the RNPP. The first one optimizes average energy cost and average sensitivity area. The second one optimizes the two previous objectives and network reliability. The authors propose to solve the two problems through a wide range of MO metaheuristics from the three main groups in the field: evolutionary algorithms, swarm intelligence algorithms, and trajectory algorithms. These algorithms are the Non-dominated Sorting Genetic Algorithm II (NSGA-II), Strength Pareto Evolutionary Algorithm 2 (SPEA2), Multi-Objective Evolutionary Algorithm based on Decomposition (MOEA/D), Multi-Objective Artificial Bee Colony (MO-ABC), Multi-Objective Firefly Algorithm (MO-FA), Multi-Objective Gravitational Search Algorithm (MO-GSA), and Multi-Objective Variable Neighbourhood Search Algorithm (MO-VNS). The results obtained are statistically analysed to determine if there is a robust metaheuristic to be recommended for solving the RNPP independently of the number of objectives.

## 1. Introduction

Over the last years, Wireless Sensor Networks (WSNs) have attracted a great interest in both industry and academy. This fact is because of the open possibilities for monitoring systems and environments with reduced deployment and maintenance costs. As a result, many practical applications were rolled out with the advancement of technologies. For instance, they were successfully applied for smart grids, smart farming, and smart buildings [1,2,3].

A Traditional Wireless Sensor Network (T-WSN) is composed of many sensors capturing information and a single sink node (or several ones) collecting all data. The sensors have some interesting features, which encourage the use of this technology. For instance, they are small, power-autonomous, wireless, cheap, and able to capture different types of measures in the same device. Moreover, the wire absence facilitates the network deployment, meaning a reduction of the deployment costs. These features, among others, allow considering WSNs in environments, where the deployment of wired technologies would be expensive or even impossible [4].

Nevertheless, WSNs also present important shortcomings, e.g., latency, coverage, energy efficiency, computing capacity, security, and network lifetime. Such factors are important for maintaining the Quality of Service (QoS), which is critical for many real-world applications [5]. One aspect of great importance in WSNs is energy efficiency. This fact is due to sensors are usually powered by batteries, whose replacement is often difficult, involving a cost and a loss of performance [6].

In case that a T-WSN considers a simple star topology, the energy cost distribution is similar for all sensors over the operation time. However, in a real scenario, the topology is quite different because of network size, application requirements, or land orography. Under these circumstances, it is usual to consider a multi-hop topology, where sensors relay data. As expected, the energy cost distribution in a multi-hop topology could be unbalanced due to the relaying task of sensors, generating bottlenecks, i.e., sensors subject to higher energy costs than others, meaning that batteries drain faster. These bottlenecks could generate disconnected areas in the WNS, negatively affecting QoS. A possible solution to this issue is the T-WSN deployment with redundant sensors. However, this focus implies high maintenance and deployment costs.

In response to the need to avoid bottlenecks in multi-hop topologies, a device specialized in communication tasks and called Relay Node (RN) was added to T-WSNs [7]. RNs send all information received to the sink node, so reducing the workload of sensors in the vicinity. There are two different approaches under this network model: Single-Tiered and Two-Tiered WSNs, ST-WSNs and TT-WSNs, respectively. The first model considers that all devices can communicate among them by following a multi-hop routing protocol. The second model is a cluster-based network, where the sensors send data to the RN in one hop; then, the RN forwards the data to the sink node in one or more hops, where the path only includes other RNs and the sink node. In both network models, RNs should have higher energy capacity than sensors, e.g., having large batteries, being plugged into the grid, or being energy-harvesting devices. As a consequence, RNs are significantly more expensive than sensors, and then their deployment should be carefully studied to maximise the investment.

The deployment of WSNs was defined as a Non-deterministic Polynomial-time hard (NP-hard) optimization problem in the literature [8,9]. As known, exact techniques are not recommended for solving NP-hard problems, due to computing time increases exponentially with the problem dimension. Instead, approximate techniques should be considered, e.g., heuristics and metaheuristics [10]. Note that it is possible to solve NP-hard problems using exact techniques for instances of limited size [11].

If the deployment problem considers a single-objective focus, the most relevant performance metric is selected as the optimization objective, while the remaining metrics are treated as constraints. This single-objective approach may be biased in real-world applications because it is supposed the importance of one metric to the detriment of others. A Multi-Objective (MO) approach means a more realistic focus, where several conflicting objectives are simultaneously optimized [12].

On this basis, this paper studies how to efficiently deploy energy-harvesting RNs in previously-established static T-WSNs by following an ST network model. This problem is known as the Relay Node Placement Problem (RNPP). To this end, the authors apply a set of MO metaheuristics to solve two formulations of the same RNPP with a different number of objectives: two and three. The goal of this analysis is to identify potential robust MO techniques, which could be recommended as a general solving-method for problems as the one considered in this work. To the best of our knowledge, this is the first work which performs this type of study for a WSN deployment problem.

The following tasks are performed through this study:

- A formal statement is provided for the two formulations of the RNPP addressed. The first formulation optimizes Average Energy Cost (AEC) and Average Sensitivity Area (ASA). The second formulation optimizes the two previous objectives and Network Reliability (NR).
- The authors apply eight MO metaheuristics specially adapted for solving the problem addressed. The algorithms are from the three main groups in the field [13]: Evolutionary Algorithms (EAs), Swarm Intelligence Algorithms (SIAs), and Trajectory Algorithms (TAs). Concretely, the authors consider three EAs: Non-dominated Sorting Genetic Algorithm II (NSGA-II) [14], Strength Pareto Evolutionary Algorithm 2 (SPEA2) [15], and Multi-Objective Evolutionary Algorithm based on Decomposition (MOEA/D) [16]. Three SIAs: Multi-Objective Artificial Bee Colony (MO-ABC) [17], Multi-Objective Firefly Algorithm (MO-FA) [18], and Multi-Objective Gravitational Search Algorithm (MO-GSA) [19]. A TA: Multi-Objective Variable Neighbourhood Search Algorithm (MO-VNS) [20]. A modified approach of MO-VNS and called MO-VNS* is also considered, as will be discussed in Section 5.
- The previously introduced MO metaheuristics are applied for solving the two formulations of the RNPP while optimizing a freely available dataset proposed by ourselves in [21]. This is because standard data sets do not exist for the problem definition. Instead, authors in the recent scientific literature consider non-public or randomly generated data sets. Thus, the results obtained in this paper could be replicated or improved by other authors in future works.
- The results obtained are analysed through an accepted statistical methodology to determine if there is any metaheuristic which provides a significantly better performance for each formulation. As a result, we could conclude if there is any metaheuristic which provides a robust performance independently of the problem complexity and the number of objectives.

The rest of this paper is structured as follows. Section 2 discusses the related work. Section 3 describes the WSN model considered. Section 4 defines the two optimization problems. Section 5 discusses the MO metaheuristics considered. Section 6 includes the solving strategy. Section 7 exposes the experimental results. Finally, conclusions and future lines of research are left for Section 8.

## 2. Background

This section reviews related works within the RNPP in WSNs, focusing on the two sets of techniques mainly considered in the literature, which are heuristics and metaheuristics.

Starting with heuristics, we may cite the following relevant works. Hou et al. [7] deployed RNs in TT-WSNs while minimizing the network geometric deficiencies and maximizing the network lifetime. Tang et al. [22] applied two polynomial time approximation algorithms to determine which was the minimum number of RNs to ensure fault-tolerance and connectivity in TT-WSNs. Wang et al. [23] optimized the network cost in TT-WSNs with constraints on the lifetime and connectivity, while considering two models: all nodes were energy limited and the RNs were not energy limited. Lloyd and Xue [24] optimized the network lifetime and preserved connectivity in ST-WSNs by following two approaches: between each pair of sensors there was a path composed of RNs and/or sensors, and on the other hand, the path included only RNs. Han et al. [25] maximized fault-tolerance in ST-WSNs while assuming sensors with different transmission radius. Xu et al. [26] studied the impact of random device placement on connectivity and lifetime in TT-WSNs. Misra et al. [27] ensured connectivity in ST-WSNs by deploying a minimum number of RNs, where the RNs were constrained to be placed at a subset of candidate locations, this is the so-called Constrained RNPP (C-RNPP). Nigam and Agarwal [28] designed a branch-and-cut algorithm to deploy the minimum number of RNs in ST-WSNs, such that there was a prespecified delay bound between the sensors and the sink node. Misra et al. [29] addressed the C-RNPP in ST-WSNs minimising the number of RNs needed, while keeping connectivity, in an energy-harvesting network, in which the energy harvesting potential of the candidate locations was known a priori. Ma et al. [30] proposed a connectivity-aware algorithm for RN placement in TT-WSNs. Concretely, the authors considered a local search approximation algorithm to solve the problem. Djenouri and Bagaa [31] proposed a heuristic method to prolong the network lifetime by deploying additional sensors and RNs in both ST and TT-WSNs by following a C-RNPP. Ranga et al. [32] proposed a method to heal the network partition problem in ST-WSNs focused on connectivity. Izadi et al. [33] proposed a fuzzy-based self-healing coverage scheme for randomly deployed mobile sensor nodes in ST-WSNs. The proposed scheme determined the uncovered sensing areas and then selected the best mobile nodes to be moved to minimize the coverage hole. Sitanayah et al. [34] proposed two algorithms (Greedy-MSP and GRASP-MSP) for solving the problem of multiple sink placement to minimise the deployment cost while ensuring that each sensor node in the network was double-covered. They also proposed two algorithms (Greedy-MSRP and GRASP-MSRP) for solving the problem of deploying sinks and RNs minimise the deployment cost and guarantee that all sensor nodes in the network were double-covered. Bagaa et al. [35] tackled the deployment of RNs in ST-WSNs by following a C-RNPP. The authors focused on minimising the outage probabilities when constructing the routing tree by adding a minimum number of RNs that guaranteed connectivity.

Following with metaheuristics, we may cite the following relevant works. Zhao and Chen [36] implemented a Particle Swarm Optimization (PSO) to minimize the energy expenditure in ST-WSNs. Perez et al. [37] assumed an MO-EA to optimize the number of RNs deployed and the energy cost in ST-WSNs by following a C-RNPP. Peiravi et al. [38] considered an MO-GA to optimize the network lifetime for several delay values in TT-WSNs. Gupta et al. [39] proposed two algorithms for relay node placement in ST-WSNs, providing k-connectivity of the sensor nodes, where the first algorithm is a Genetic Algorithm (GA) and the second one is based on a greedy approach. Hashim et al. [40] proposed an enhanced deployment algorithm based on Artificial Bee Colony (ABC) to extend the lifetime in TT-WSNs. George and Sharma et al. [41] considered a GA to deploy RNs in ST-WSNs by following a C-RNPP approach to minimise the number of RNs while providing maximum connectivity. Yu et al. [42] studied how to deploy RNs in ST-WSNs while optimizing energy cost and reliability, using to this end three MO metaheuristics.

The proposal in this work differs from the papers introduced before in the following: (i) the network model considers a Single-Tiered (ST) approach, which is usual in medium-size networks assuming low-cost devices, such as occurs in intensive agriculture [43]. (ii) In this model, the RNs are similar to sensors, but without having electronics for capturing data, instead, they are specialized in communication. Moreover, RNs have harvested capabilities and then, they can be deployed without taking into account the existence of power sources, such as plugs. This fact results in an unconstrained RNPP, meaning that the search space is greater than in the C-RNPP focus. (iii) We address the RNPP through MO metaheuristics, providing a trade-off between the conflicting objectives, not as with heuristics, which is useful in the decision-making of network designers. As introduced before, many heuristics were proposed for addressing the ST-RNPP [24,25,27,28,29,31,32,33,35]. However, only a few papers considered metaheuristics to this end [36,37,39,41,42]. Regarding these latest works, the authors in [36,39,41] considered a single-objective focus and our proposal is MO. The authors in [37] considered a MO focus for the number of NRs and energy cost, but following a C-RNPP. Finally, the authors in [42] considered a similar focus to ours, but assuming three MO metaheuristics and an optimization problem with two objectives (reliability and energy cost). In our proposal, we consider a wide range (seven) of MO metaherustics to solve two formulations of the RNPP, with two and three objectives, to identify a robust metaheuristic to solve the problem.

This work is partially inspired by some early papers. The bi-objective RNPP was addressed in [44,45,46] for NSGA-II, SPEA2, MO-ABC, MO-GSA, MO-VNS, and MO-VNS*. The three-objective RNPP was addressed in [47] for NSGA-II, SPEA2, MOEA/D, MO-ABC, MO-VNS, and MO-FA. This paper presents a more complete development with an intensive statistical study comparing the performance of the metaheuristics solving the two RNPPs. As expected, this work includes experimental results never published before, such as the performance of MO-FA and MOEA/D solving the bi-objective problem and MO-GSA and MO-VNS* for the three-objective case.

## 3. Network Model

This section describes the WSN model considered, including general assumptions and specific details about energy cost, sensitivity area, network lifetime, and network reliability. For clarity, the mathematical notation used in this work is described in the abbreviation part of this document.

### 3.1. General Assumptions of the WSN Model

1. The network is composed of a sink node, ${\tilde{s}}_{s}$ sensors, and ${\tilde{s}}_{r}$ RNs, which can be linked following an ST approach if they are at a distance lower than the communication radius $r_{c}$. All these devices are placed on a same outdoor unrestricted 2D-surface with size $d_{x}\times d_{y}$, where there is not any relevant obstacle nor external interference.
2. Sensors are powered by batteries. The sink node and RNs are energy-harvesting devices, having enough energy capacity for operating over network lifetime.
3. Initially, at time t=0, all sensors start with the same energy capacity iec in the batteries. If a sensor is exhausted during operation (t>0), it cannot be linked again, i.e., lifetime after battery replacement is not considered.
4. Sensors capture information about the environment on a regular basis with a sensitivity radius $r_{s}$, i.e., a sensor covers a circumference of radius $r_{s}$. All information captured is immediately sent to the sink node.
5. The sink node is the only connection point of the network to the outside.
6. RNs are low-cost devices with a similar conception that sensors, but without capturing data. Thus, they only send all the information received to the sink node.
7. RNs have the necessary computational resources to manage network traffic while maintaining a low-power consumption to facilitate the harvesting design and reduce the cost of the solution. This fact is usually addressed by using a low-power microprocessor, which generally has a higher computing capacity than the one used in the sensors.
8. The routing protocol for all devices is the one provided by Dijsktra’s Algorithm [48] for minimum path length among devices.
9. A perfect synchronization among devices and an efficient MAC protocol are supposed, reducing energy cost because of retransmissions and idle time.

### 3.2. Energy Cost

We consider the energy model proposed by [49] to simulate the energy cost of sensors during operation time. Note that sensors are the only devices which are subject to energy limitations in our model. In this formulation, a sensor $i=\left(x,y\right)\in S_{s}\left(t\right)$ where $x\in [0,d_{x}]$ and $y\in [0,d_{y}]$ sends a number of packets $P_{i}\left(t\right)$ at time t>0 given by

(1) $$P_{i}\left(t\right)=1+Rp_{i}\left(t\right).$$

This formulation considers the number of packets captured by *i*, a packet per instant time, and the number of packets relayed by the sensor *i*, $R_{p_{i}}\left(t\right)$, because of the multi-hop routing protocol, which is given by

(2) $$Rp_{i}\left(t\right)=\sum_{j\in \{S_{s}\left(t\right)-i\}}z_{j,i}^{c}\left(t\right),$$

where $z_{j,i}^{c}\left(t\right)$ is a variable assuming 1 if $i\in S_{s}\left(t\right)$ is in the minimum path between $j\in S_{s}\left(t\right)$ and the sink node *c* at t>0, and 0 otherwise.

The energy cost $Ee_{i}\left(t\right)$ of a sensor *i* at time *t* is given by

(3) $$Ee_{i}\left(t\right)=P_{i}\left(t\right)\;\beta \;amp\;k\;(||i-w_{i}^{c}{\left(t\right)||}_{d}{)}^{\alpha },$$

where $\left|\right|\cdot {\left|\right|}_{d}$ is the Euclidean distance between two points, β>0 is the transmission quality parameter, amp>0 is the energy cost per bit of the power amplifier, k>0 is the information packet size in bits, $w_{i}^{c}\left(t\right)$ is the variable providing the next device in the minimum path between $i\in S_{s}\left(t\right)$ and the sink node at time t>0, and α∈[2,4] is the path loss exponent. Thus, the energy charge $Ec_{i}\left(t\right)$ of a sensor *i* at time *t* is given by

(4) $$Ec_{i}\left(t\right)=\left\{\begin{array}{cc} Ec_{i}\left(t-1\right)-Ee_{i}\left(t\right) & \;\mathrm{if}\;t>0\\ iec & \;\mathrm{if}\;t=0 \end{array},\right.$$

where iec denotes the initial energy charge of the sensors, for iec>0. Hence, if $Ec_{i}\left(t\right)$ equals zero, the sensor is out of energy, and then it cannot be linked. Otherwise, it is active.

### 3.3. Sensitivity Area

As stated before, a sensor covers a circumference of radius $r_{s}$ and area $\pi r_{s}^{2}$. Therefore, at time *t*, the WSN sensitivity area is calculated as the union of the areas of the active sensors at time *t* with a path to the sink node, $s_{s}\left(t\right)$. The intersection calculation is a known complex problem, where computational effort increases exponentially with the number of circles [50]. As a usual approach to approximate this calculation, the set of binary demand points ${\tilde{D}}_{p}\left(t\right)$ is uniformly distributed on the surface [51,52,53]. Then, the number of demand points ${\tilde{d}}_{p}\left(t\right)$ with an active sensor at a distance lower than $r_{s}$ are counted. According to this approximation, the sensitivity area A(t) provided by a WSN at time t>0 is given by

(5) $$A\left(t\right)=\frac{1}{{\tilde{d}}_{p}\left(t\right)}\sum_{p\in {\tilde{D}}_{p}\left(t\right)}a_{p}\left(t\right),$$

where $a_{p}\left(t\right)$ is the indicator function defined as

(6) $$a_{p}\left(t\right)=\left\{\begin{array}{cc} 1 & \;\mathrm{if}\;\;\exists i\in S_{s}\left(t\right):||p-{i||}_{d}<r_{s}\\ 0 & \;\mathrm{otherwise} \end{array}\right.,$$

i.e., $a_{p}\left(t\right)$ equals 1 if there is an active sensor *i* at a distance lower than $r_{s}$ from the demand point *p*.

Within this approach, we consider that demand points follow a grid distribution with a distance between neighbouring points of $d_{pn}$. Hence, the number of demand points at time t>0 is

(7) $${\tilde{d}}_{p}\left(t\right)=\frac{d_{x}\;d_{y}}{d_{pn}}.$$

### 3.4. Network Lifetime

The network lifetime $t_{n}$ is defined as the number of time periods over which the information provided by the network is useful for an application. We formulate this concept based on a threshold sensitivity area $co_{th}$, that is

(8) $$t_{n}=\left|\right|\left\{t>0\in \tau :A\left(t\right)>co_{th}\right\}\left|\right|,\;\mathrm{for}\;\tau =0,1,2,\ldots ,$$

where ||·|| is the cardinal of a set and τ is the set of time periods.

### 3.5. Network Reliability

We consider the network reliability formulation in [54], which is defined based on the number of disjoint paths between a given sensor and the sink node through Suurballe’s Algorithm [55]. Thus, the reliability $re_{i}$ of a sensor *i* of the initial sensor sets ${\tilde{S}}_{s}$ is

(9) $$re_{i}=1-\prod_{l=1}^{djp_{i}^{c}}\left(1-{\left(1-err\right)}^{h_{l}^{i,c}}\right),$$

where $djp_{i}^{c}$ denotes the number of disjoint paths between the sensor $i\in {\tilde{S}}_{s}$ and the sink node, err∈[0,1] is the local channel error, and $h_{l}^{i,c}$ is the number of hops in the *l*-th disjoint path between $i\in {\tilde{S}}_{s}$ and the sink node.

## 4. Optimization Problems

Let $f_{1}\in R^{+}$ be the AEC of the sensors over the network lifetime defined as

(10) $$f_{1}=\frac{1}{t_{n}}\sum_{t=1}^{t_{n}}\left(\sum_{i\in S_{s}\left(t\right)}\frac{Ee_{i}\left(t\right)}{s_{s}\left(t\right)}\right).$$

This objective is related to the energy efficiency problem in WSNs [56], whose goal is to reduce energy cost while balancing energy distribution and increasing network lifetime.

Let $f_{2}\in \left[0,1\right]$ be the ASA provided by the WSN over the network lifetime defined as

(11) $$f_{2}=\frac{1}{t_{n}}\sum_{t=1}^{t_{n}}A\left(t\right).$$

This objective is related to the coverage problem in WSNs [57], whose goal is to optimize diversity and the amount of information provided by the network.

Let $f_{3}\in \left[0,1\right]$ be the NR based on the connectivity of sensors defined as

(12) $$f_{3}=\frac{1}{{\tilde{s}}_{s}}\sum_{i\in {\tilde{S}}_{s}}re_{i}.$$

This objective is related to the reliability problem in WSNs [58], whose goal is to get trustable networks.

On this basis, the authors define the bi-objective optimization problem as follows. Given a previously deployed T-WSN, i.e., ${\tilde{s}}_{s}$ initial sensors and a sink node, the objective is to deploy ${\tilde{s}}_{r}$ RNs assuming an ST network model to

(13) $$\min f_{1},\max f_{2},$$

subject to

(14) $$\forall r\in {\tilde{S}}_{r},\;r=\left(x,y\right):x\in \left[0,d_{x}\right]\;\mathrm{and}\;y\in \left[0,d_{y}\right].$$

That means, all the RNs deployed are in the limits of the scenario.

The three-objective optimization problem is similar to the bi-objective one stated before, but including an additional objective, while maintaining the same constraints. That is, the goal is to

(15) $$\min f_{1},\max f_{2},\max f_{3}.$$

Note that $f_{1}$, $f_{2}$, and $f_{3}$ are conflicting with each other as shown in [47]. As is well-known, this fact is a fundamental requirement, which any MO optimization problem should fulfil.

## 5. Metaheuristics

This section describes the MO metaheuristics considered for solving the two problems. As introduced before, some of the metaheuristics were exposed in prior works by ourselves and then, we chose not to include the whole implementing information in the present proposal to avoid duplicity. Instead, we next detail some key aspects adapting the meheuristics to solve the problem, for further information we recommend readers going to the specific works [44,45,46,47]:

- NSGA-II: It considers two populations $P_{n}^{g}$ and $Q_{n}^{g}$ with $ps_{n}$ individuals each, where $P_{n}^{g}$ saves the parents of the current iteration *g* and $Q_{n}^{g}$ saves the offspring generated based on $P_{n}^{g}$. The individuals in the populations follow the same chromosome structure, where each individual has many genes as RNs should be deployed in the solution. Note that a gene includes the 2D-coordinates of an RN. This structure is considered for the remaining algorithms exposed in this section. Each individual in $Q_{n}^{g}$ is generated by applying crossover and mutation operators based on two previously selected solutions from $P_{n}^{g}$. The crossover operator is the usual one-point-crossover with a crossover probability $cro_{n}$. The mutation operator applies random changes in the genes of the solution generated by the crossover operator according to a mutation probability $mut_{n}$. The populations for the next iteration are generated as follows. $P_{n}^{g+1}$ is generated by selecting the best $ps_{n}$ solutions combining $P_{n}^{g}$ and $Q_{n}^{g}$ according to the crowded-comparison operator ${≺}_{n}$ [14] and $Q_{n}^{g+1}$ is initialized to empty.
- SPEA2: It considers a regular population $P_{s}^{g}$ of size $ps_{s}$ and an auxiliary population ${\bar{P}}_{n}^{g}$ of size ${\bar{ps}}_{s}$, saving the best individuals found so far. The methodology followed by SPEA2 is similar to NSGA-II, but considering a different selection strategy to ${≺}_{n}$ when generating $P_{s}^{g+1}$. We consider the same crossover and mutation operators as for NSGA-II with probabilities $cro_{s}$ and $mut_{s}$.
- MO-ABC: It is an MO approach of ABC, which was adapted by ourselves based on the ${≺}_{n}$ concept. The algorithm considers a population $P_{a}^{g}$ with size $ps_{a}$. The parameter $se_{a}$ determines the percentage of solutions in $P_{a}^{g}$ managed by employed forager bees and the remaining ones are managed by onlooker bees. An employed forager bee tries to improve the solution by looking in its surrounding, i.e., RN coordinates are lightly modified. Note that the new solution is only accepted if it is better in fitness value; otherwise, it is discarded. If an employed forager bee tries to improve the solution $limit_{a}$ times without any improvement in fitness value, then the solution is supposed exhausted, being mandatory be managed by an scout bee. An onlooker bee tries to improve the solution by looking in the surrounding of a randomly selected employed forager bee. As before, the solution is only accepted it it is better in fitness value. An scout bee generates a new solution based on a randomly selected onlooker bee solution from the two first Pareto fronts in $P_{a}^{g}$. Next, the Euclidean distance between the solution selected and all other solutions in $P_{a}^{g}$ is calculated. The new solution is obtained by combining the $k_{a}$-nearest solutions to the selected one, being $k_{a}$ a random value in 2,…,11. On the contrary that for employed forager and onlooker bees, the solution generated by the scout bee is directly accepted without analyzing the improvement in fitness value. $P_{a}^{g+1}$ is generated by including the solutions generated by the corresponding bees.
- MO-FA: It is an MO approach of the Firefly Algorithm (FA), which was adapted by ourselves based on the ${≺}_{n}$ concept. In this algorithm, a firefly is a possible solution to the problem and its brightness is defined by its solution quality. The attractiveness that a brighter firefly causes in a less bright one implies a movement in its RNs controlled by $r_{f}\in \left[0,1\right]$, ${\beta_{0}}_{f}\in \left[0,1\right]$, and $\lambda_{f}\in \left(0,\infty \right)$ parameters. Thus, MO-FA considers two populations $P_{f}^{g}$ and $Q_{f}^{g}$ with size $ps_{f}$, where $P_{f}^{g}$ saves the fireflies at the beginning of the iteration *g* and $Q_{f}^{g}$ contains the resulting fireflies after applying the attractiveness mechanism in $P_{f}^{g}$. The populations for the next iterations are generated as follows. $Q_{f}^{g+1}$ is initialized to empty and $P_{f}^{g+1}$ is generated by selecting the $ps_{f}$ best solutions combining $P_{f}^{g}$ and $Q_{f}^{g}$. Finally, in case that the percentage of non-dominated solutions in $Q_{f}^{g}$, regarding $P_{f}^{g}$ is lower than $when\_sc_{f}$ ∈[0,1], then the mutation operator discussed for NSGA-II is applied with mutation probability $mut_{f}$ to each solution in $P_{f}^{g+1}$.
- MO-VNS: It considers two populations $P_{v}^{g}$ and $S_{v}^{g}$ with unlimited size, where $P_{v}^{g}$ keeps only non-dominated solutions and $S_{v}^{g}$ saves the solutions from $P_{v}^{g}$ considered to explore the search space during the current iteration *g*, i.e., $S_{v}^{g}$ is put to empty at the beginning of the iteration. A non-considered solution is selected from $P_{v}^{g}$ until all solutions were selected. The solution is used to generate new individuals in its surrounding based on $neigh_{v}$ neighbourhood structures. Each structure determines how different could be the new solution compared to the initial one in terms of maximum displacement of the RNs, which is limited by the $ns_{v}\in \left[1,\infty \right)$ parameter. Thus, the neighborhood structures are iteratively applied from higher to lower displacement, generating new solutions to be included in $P_{v}^{g+1}$ if they fulfill the non-dominated requirement.
- MO-VNS*: It considers the same focus as for MO-VNS but including a perturbation mechanism at the end of each iteration. This mechanism is performed for each solution in $P_{v}^{g+1}$ by applying the mutation operator discussed for NSGA-II and SPEA2 with mutation probability $mut_{v}$.
- MO-GSA: It is an MO approach of the Gravitational Search Algorithm (GSA), which was adapted by ourselves based on the ${≺}_{n}$ concept. In this algorithm, an object is a possible solution to the problem and its mass is defined by its solution quality. All objects are mutually attracted by the Newtonian gravity force, causing a global movement of all objects towards heavier masses, corresponding the better solutions. The algorithm considers two populations $P_{ga}^{g}$ and $Q_{ga}^{g}$ with $ps_{ga}$ individuals, where $P_{ga}^{g}$ saves the objects at the beginning of *g*, before acting gravitational forces, and $Q_{ga}^{g}$ contains the resulting objects after applying the forces in $P_{ga}^{g}$. $P_{ga}^{g+1}$ is generated by selecting the best $ps_{ga}$ solutions combining $P_{ga}^{g}$ and $Q_{ga}^{g}$. Finally, in case that the percentage of non-dominated solutions in $Q_{ga}^{g}$, regarding $P_{ga}^{g}$ is lower than $when\_sc_{ga}$, then the mutation operator for NSGA-II is applied with mutation probability $mut_{ga}$ to each solution in $P_{ga}^{g+1}$.
- MOEA/D: It decomposes an MO optimization problem into several single-objective sub-problems by distributing reference points on the Convex Hull of Individual Minima (CHIM), based on the NBI-Tchebycheff approach [59]. The distribution is performed according to $CHIMinc_{m}\in \left[1,\infty \right)$ and $crow_{m}\in \left(0,\infty \right)$ parameters, where $CHIMinc_{m}$ defines how the extreme points of the CHIM are reassigned to increase the search area and $crow_{m}$ defines the distance between two any reference points. Each reference point is assigned a set of reference points in its neighbouring according to the Euclidean distance, where the cardinal of this set is given by the $neigh_{m}$ parameter. On this basis, MOEA/D considers a regular population $P_{m}^{g}$, where each individual is associated with a different reference point in the CHIM, and an auxiliary population $F_{m}^{g}$ of undefined size. Thus, $P_{m}^{g}$ contains the individuals considered to generate solutions in the *g*-th iteration and $F_{m}^{g}$ saves the non-dominated solutions found until *g*. Over generations and for each solution in $P_{m}^{g}$, two neighbouring solutions are selected (based on the neighbouring structure previously generated) to then produce a solution based on the crossover and mutation operators defined for NSGA-II, with $cro_{m}$ and $mut_{m}$, respectively. The solution generated replaces the previous one only if it is better in fitness value for the corresponding single-objective sub-problem.

Some of the previously exposed metaheuristics were not considered in prior works for solving any of the two problems addressed in the present proposal. These algorithms are:

- MO-FA and MOEA/D for the bi-objective approach.
- MO-GSA and MO-VNS* for the three-objective approach.

Without loss of generality, MO-FA, MO-GSA, and MO-VNS* can be implemented with minimal changes independently of the number of objectives. Most changes are related to the implementation of ${≺}_{n}$ as detailed in [45,46,47].

On the contrary, the implementation of MOEA/D for the bi-objective case requires further explanation. Suppose a bi-objective optimization problem maximizing $f_{1}$ and $f_{2}$. Let $F_{1}=\left(\mathrm{maxF}\left(f_{1}\right),\mathrm{minF}\left(f_{2}\right)\right)$ and $F_{2}=\left(\mathrm{minF}\left(f_{1}\right),\mathrm{maxF}\left(f_{2}\right)\right)$ be the two extreme points delimiting the objective space, where maxF(·) and minF(·) denote the upper and lower bounds of a fitness function. Let $\Upsilon =\{r^{1},\ldots ,r^{ps_{m}}\}$ be a set of points evenly distributed on the plane I, for $r^{i}=\left(r_{1}^{m},r_{2}^{m}\right)\in \Upsilon$, $m\in 1,\ldots ,ps_{m}$, where $ps_{m}$ is the cardinal of Υ and $F_{1},F_{2}\in \Upsilon$. Let $\vec{v_{n}}=(n_{1},n_{2}$) be a normal vector to the plane I. On this basis, the bi-objective optimization problem is decomposed into $ps_{m}$ single-objective minimization subproblems, where the *m*-th subproblem optimizes the function $g(x:r^{m},\vec{v_{n}})$ given by

(16) $$g\left(x:r^{m},\vec{v_{n}}\right)=\max\left\{n_{1}\;\left(f_{1}\left(x\right)-r_{1}^{m}\right),n_{2}\;\left(f_{2}\left(x\right)-r_{2}^{m}\right)\right\},$$

where *x* is a solution to the optimization problem.

In addition of how to decompose the optimization problem, another important aspect in MOEA/D is the distribution of the reference points Υ in the plane I. Algorithm 1 shows the procedure considered for distributing such points for the bi-objective approach by following a straight line defined by $F_{1}$ and $F_{2}$. This algorithm considers as input $F_{1}$, $F_{2}$, $crow_{m}$, and $CHIMinc_{m}$. Initially, at step 0 (lines 1–3), the new extreme points $E_{1}$ and $E_{2}$ depending on $F_{1}$ and $F_{2}$ are calculated solving the expression given by

(17) $$d\left(E_{1},E_{2}\right)=d\left(F_{1},F_{2}\right)\;CHIMinc_{m},$$

where d(·) provides the Euclidean distance between two points. At step 1 (line 4), the number of divisions $n_{E_{1},E_{2}}$ in the segment $\bar{E_{1}E_{2}}$ is calculated. Finally, at step 2 (lines 5–8), reference points in the segment $\bar{E_{1}E_{2}}$ are generated. From this point, MOEA/D can be implemented with minimal changes independently of the number of objectives as detailed in [47].

**Algorithm 1** Distribution of the reference points for two objectives.
1:
Υ←{}
2:
$ps_{m}\leftarrow 0$
3:
$\left(E_{1},E_{2})\leftarrow \mathrm{scaleExtremePointsCHIM}(F_{1},F_{2},CHIMinc_{m}\right)$
4:
$n_{E_{1},E_{2}}\leftarrow d\left(E_{1},E_{2}\right)/crow_{m}$
5:
**for** $m\leftarrow 0\;\mathrm{to}\;n_{E_{1},E_{2}}$ **do**
6: $\Upsilon \leftarrow \Upsilon \cup \{E_{1}+m\;\left(E_{2}-E_{1}\right)/\left(n_{E_{1},E_{2})}\right\}$7: $ps_{m}\leftarrow ps_{m}+1$8:
**end for**



## 6. Solving Strategy

This section discusses the dataset used for analyzing the algorithms, the experimental methodology, and the parametric sweep task.

### 6.1. Dataset Description

We consider the freely available dataset in [21] composed of four scenarios. For each of them a T-WSN was deployed considering that (i) the sink node was placed on the middle of the surface and (ii) the number of sensors was the lower bound to cover the whole surface defined as

(18) $$\tilde{s_{s}}=\frac{d_{x}\;d_{y}}{\pi \;r_{s}^{2}}.$$

In this dataset, two $r_{c}$ values, 30 m and 60 m, are considered to simulate different communication conditions. Hence, two homogeneous instances are defined for each scenario following the notation $d_{x}\;\times \;d_{y}\_r_{c}$. Table 1 shows additional details about the dataset, including scenario size, fitness values without deploying any RN ($\tilde{s_{r}}=0$) for both $r_{c}$ values, hypervolume reference points, and test cases (RNs to be deployed using the metaheuristics, while keeping a ratio of number of devices to RNs lower than 20%). Note that for $r_{c}=60$ m, reliability is not necessary to optimize because the number of disjoint paths is high between any sensor and sink node. Thus, instances with $r_{c}=60$ m will not be optimized using the three-objective approach. The remaining parameters of the network model take values α=2.00, β=1.00, $co_{th}=0.70$, k=128 KB, $r_{s}=15$ m, iec=5 J, and amp=100 pJ/bit/m${}^{2}$ from the literature [51,60].

### 6.2. Experimental Methodology

The dataset in Section 6.1 is optimized using the previously introduced metaheuristics for each of the two optimization problems. To this end, we perform 31 independent runs for each metaheuristic, instance, test case, and optimization problem. Five stop conditions are considered based on the number of evaluations to study convergence, i.e., 50,000, 100,000, 200,000, 300,000, and 400,000 evaluations. The results obtained are evaluated using hypervolume and set covering.

### 6.3. Parametric Sweep

The metaheuristics were configured for solving each problem as follows. (i) Starting from default values, (ii) a parameter of the algorithm is selected to be tuned. (iii) Then, 31 independent runs are performed for each value of the parameter in a range, while the others remain fixed. (iv) The configuration providing the best average behavior based on hypervolume is selected, overwriting the default parameter value. (v) Next, a non-configured parameter is selected going to step (ii). This configuring methodology ends when all parameters were selected in step (v). Table 2 shows the configurations selected (*2Obj* and *3Obj* fields for the bi-objective and three-objective approach, respectively) and the range studied.

## 7. Experimental Results

This section includes the experimental results obtained by solving both the bi-objective and the three-objective RNPPs.

### 7.1. Bi-Objective Approach

Table 3 shows average hypervolume for MO-VNS*, MO-ABC, MO-VNS, MO-FA, MO-GSA, and MOEA/D, solving the bi-objective RNPP for each test case and stop condition. In this table, hypervolumes in bold correspond to results never before published. Note that the hypervolumes for NSGA-II and SPEA2 were not shown to simplify the table because they reported significantly lower hypervolumes than the other algorithms, instead, we refer readers to [46]. If we analyze how hypervolumes change over stop conditions, we check that most algorithms show an homogeneous growth, reaching an asymptotic trend for 400,000 evaluations. That means that the set of stop conditions selected is representative to study convergence. On the other hand, if we analyze the tables focusing on shaded cells showing higher (better) hypervolumes, we note that some algorithms seem to outperform others. To check if the differences are significant, we analyze the data using the statistical methodology as follows.

First, we remove possible outliers from the hypervolume distributions. Next, we analyze if data follow a normal distribution or not through Kolmogorov-Smirnov-Lilliefor’s and Shapiro-Wilk’s tests with hypothesis $H_{0}$: data follow a normal distribution and $H_{1}$: otherwise. As both tests provided p-values lower than 0.05 for all cases, we should consider a non-parametric test to compare the algorithms two by two. Specifically, as samples are independent, we consider Wilcoxon-Mann-Whitney’s test with hypothesis $H_{0}$: $\bar{Hyp_{i}}\leq \bar{Hyp_{j}}$, ∀i,j∈{1=NSGA,2=SPEA2,3=MO−VNS,4=MO−VNS*,5=MO−ABC,6=MO−FA,7=MO−GSA,8=MOEA/D}, i≠j and $H_{1}$: $\bar{Hyp_{i}}>\bar{Hyp_{j}}$. Table 4 shows the percentage of test cases where a metaheuristic is significantly better than any other based on the p-values obtained before analyzed with a significance level of 0.05 and according to instance size.

Analyzing Table 4, for 50×50 instances, we check that MO-ABC, MO-FA, MO-GSA, and MOEA/D provide a similar behavior with up to 9.71%, followed by MO-VNS and MO-VNS* with up to 6.31% and 4.85%, respectively. For 100 × 100 instances, MO-VNS provides the best behavior with up to 11.37%, followed by MO-FA and MO-ABC with up to 9.51% and 9.41%, respectively. For 200 × 200 instances, MO-FA provides the best behavior with up to 10.77%, followed by MO-VNS and MO-VNS* with up to 10.03%. For 300 × 300 instances, MO-FA provides the best behavior with up to 13.71% with up to 13.71%, followed by MO-ABC and MO-VNS* with up to 8.75% and 7.45%, respectively. For all instances, MO-FA provides the best behavior with up to 11.49%, followed by MO-ABC and MO-VNS* with up to 8.88% and 8.40%, respectively. From this hypervolume analysis, we conclude that MO-FA provides the best behavior solving the problem in general. Focusing on instance size, MO-FA is also the best algorithm solving small and large instances (50 × 50, 200 × 200, and 300 × 300) and MO-VNS is better suited for medium size instances (100 × 100).

In Table 4, the behavior of MO-VNS and MO-VNS* is significantly different although their focus is almost the same, i.e., MO-VNS is better for small instances, while MO-VNS* is better for large ones. This fact is due to, for small instances, the perturbation mechanism in MO-VNS* penalizes the number of evaluations available for exploitation, while search space is not of concern, resulting in a better performance of MO-VNS. On the other hand, the perturbation mechanism in MO-VNS* is useful for exploring bigger search spaces in large instances, without being of concern the number of evaluations consumed by the process, resulting in a better performance of MO-VNS*.

Table 5 shows the average set coverage metric of an algorithm compared to the others according to instance size. The metric was calculated using the median Pareto front obtained for each algorithm solving a given instance. Analyzing this table, for 50×50 instances, we check that MO-ABC, MO-FA, and MOEA/D provide a similar behavior with up to 100.00%, followed by MOEA/D and MO-VNS with up to 90.00% and 88.28%, respectively. For 100×100 instances, MO-VNS provides the best behavior with up to 87.56%, followed by MO-VNS* and MO-FA with up to 83.11% and 75.93%. For 200×200 instances, MO-FA provides the best behavior with up to 77.52%, followed by MO-VNS* and MO-VNS with up to 68.92% and 66.62%. For 300×300 instances, MO-FA provides the best behavior with up to 86.47%, followed by MO-VNS* and SPEA2 with up to 51.86% and 45.46%, respectively. For all instances, MO-FA is the best algorithm with up to 81.47%, followed by MO-VNS* and MO-ABC with up to 63.82% and 58.92%. From this set coverage analysis, we reach similar conclusions as for hypervolume. MO-FA provides the best behavior solving the problem in average term. If we focus on instance size, MO-VNS is the best algorithm for medium size instances and MO-FA is better suited for small and large ones.

### 7.2. Three-Objective Approach

Table 6 shows average hypervolume for MO-VNS*, MO-VNS, MO-FA, MO-GSA, and MOEA/D, solving the three-objective RNPP for each test case and stop condition. In this table, hypervolumes in bold correspond to results never before published. Note that the hypervolumes for NSGA-II, SPEA2, and MO-ABC were not shown to simplify the table because they reported significantly lower hypervolumes than the other algorithms, instead, we refer readers to [47].

Analyzing Table 6, we verify that the algorithms show a homogeneous growth with an asymptotic trend in 400,000 evaluations, as for the bi-objective approach. Hence, the stop condition is representative to analyze the performance of the algorithms. Higher hypervolumes in Table 6 are shaded, reaching that some algorithms seem to outperform others. The differences observed are analyzed following the same methodology as for the bi-objective study. After removing possible outliers, we checked that data do not follow a normal distribution, and then we considered the same hypothesis as before for the Wilcoxon-Mann-Whitney’s test. As a result, Table 7 shows the percentage of test cases where a metaheuristic is significantly better than any other according to the p-values obtained with a significance level of 0.05.

Analyzing Table 7, for 50×50 instances, we check that MO-FA provides the best behavior with up to 13.36%, followed by MOEA/D and MO-ABC with up to 9.92% and 9.54%, respectively. For 100×100 instances, we check that MO-VNS provides the best behavior with up to 11.55%, followed by MO-VNS* and MO-FA with up to 10.92% and 8.61%, respectively. For 200×200 instances, we check that MO-FA provides the best behavior with up to 12.50%, followed by MOEA/D and SPEA2 with up to 11.00% and 7.00%, respectively. For 300×300 instances, we check that MO-FA provides the best behavior with up to 11.90%, followed by MOEA/D and MO-ABC with up to 11.17% and 8.46%, respectively. For all instances, MO-FA provides the best behavior with up to 11.62%, followed by MOEA/D and MO-VNS with up to 9.74% and 6.37%. From this analysis based on hypervolume, we check that MO-FA is the best algorithm in general. For small and large instances, MO-FA is also the best algorithm. For medium size instances, MO-VNS is the best algorithm.

Table 8 shows the average set coverage metric of an algorithm compared to the others for the median Pareto front. Analyzing this table, for 50×50 instances, MO-GSA is the best algorithm with up to 81.78% followed by MO-FA and MO-VNS* with up to 72.00% and 67.86%, respectively. For 100×100 instances, MO-VNS* is the best algorithm with up to 73.19%, followed by MO-VNS and MO-FA with up to 72.36% and 65.68%, respectively. For 200×200 instances, MO-FA is the best algorithm with up to 83.71%, followed by MO-VNS* and MO-VNS with up to 49.37% and 48.77%, respectively. For 300×300 instances, MO-ABC is the best algorithm with up to 63.12%, followed by MO-FA and MO-VNS with up to 51.12% and 40.80%, respectively. For all instances, MO-FA is the best algorithm with up to 67.52% followed by MO-VNS and MO-VNS* with up to 50.80% and 49.61%, respectively. From this study, we check that MO-FA is the best algorithm on average term, followed by MO-VNS and MO-VNS*. Focusing on instance size, MO-GSA is the best algorithm for small instances, MO-VNS* is the best algorithm for medium size instances, and MO-FA is the best algorithm for large instances.

### 7.3. Bi-Objective vs. Three-Objective Approaches

**Figure 1:**
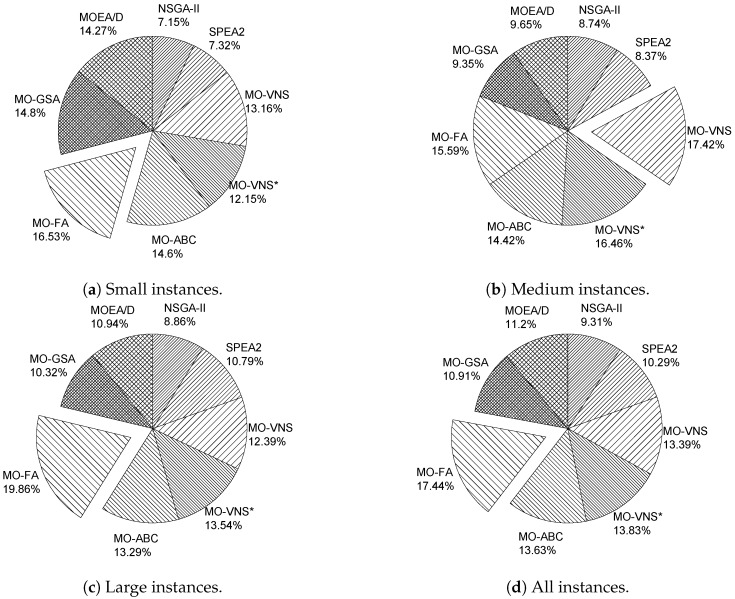
Average performance of the metaheuristics solving the two RNPPs.

Comparing the results obtained for the bi-objective and three-objective approaches in the two previous subsections, we verify that MO-FA could be recommended as a general solving method for the two approaches studied. Focusing on instance size, MO-FA provides robust performance in small and large instances, while MO-VNS provides robust performance in medium ones. This conclusion is supported by Figure 1, which shows the average performance for each metaheuristic according to the set of instances addressed. This figure was generated by combining hypervolume and set coverage values through a 1–2 standardization for the case studied. Thus, Figure 1a–d show the average performance of the metaheuristics for small, medium, large, and all instances.

The difference in performance between these two metaheuristics, as well as with the others, could be due to the movement operator used in MO-FA. This operator successfully fits the RNPP, defining a way of producing new solutions by moving the RNs in an individual according to promising solutions, resulting in that the population evolves towards better solutions. This is the reason why MO-FA provides a robust performance independently of the instance size.

For the case of MO-VNS, the algorithm defines a successfully way of generating new solutions based on an incremental local search, which is a search in the vicinity. This local search fits the RNPP, defining a way of improving a previous solution by boundedly moving the RNs. However, the performance of MO-VNS is mainly due to the application of this local search. Thus, MO-VNS is less competitive to other algorithms in large instances because the search space is large and the algorithm fails in exploration. For the case of small instances, MO-VNS is also less competitive because the search space is reduced and then, it is relatively simple for a metaheuristic to find good solutions to the problem. As a result, MO-VNS provides good performance in medium instances.

## 8. Final Remarks

This work addresses the RNPP in WSNs from two MO formulations with the purpose of searching for robust methods solving this deployment problem within a realistic perspective. The first formulation includes two objectives, energy cost and average sensitivity, and the second formulation includes three objectives, the two previous ones as well as network reliability. On this basis, the authors propose to study how performs a wide range of MO metaheuristics from the three main groups in the field: evolutionary algorithms (NSGA-II, SPEA2, and MOEA/D), swarm intelligence algorithms (MO-ABC, MO-FA, and MO-GSA), and trajectory algorithms (MO-VNS and MO-VNS*).

The eight MO metaheuristics were applied for solving four deployment scenarios, in both optimization problems, of increasing complexity, while considering a different number of RNs and communication conditions. The experimental results were analyzed through an accepted statically methodology, where two standard MO metrics were considered, i.e., hypervolume and set coverage. As a result, we concluded that MO-FA provided a robust performance independently of the number of objectives and instance size, and then MO-FA could be recommended as a general solving method for this problem. Additionally and focusing on instance size, we concluded that MO-FA provided a robust performance in small and large instances, while MO-VNS provided the best performance in medium instances.

As future lines of research, it could be interesting to extend the network model considered. For instance, simulating additional MAC and routing protocols. Moreover, it could be interesting to try to extend the results obtained to a real WSN deployment.

## Figures and Tables

**Table 1:** Dataset description.

Scenario ($d_{x}\times d_{y}$)	Fitness Values ($\tilde{s_{r}}=0,r_{c}=30$)			Fitness Values ($\tilde{s_{r}}=0,r_{c}=60$)		Hyp. Reference Points (ideal,nadir)			Test Cases ($\tilde{s_{r}}>0$)
	$f_{1}$	$f_{2}$	$f_{3}$	$f_{1}$	$f_{2}$	$f_{1}$	$f_{2}$	$f_{3}$	
50×50	0.0353	0.9175	0.9964	0.0353	0.9175	(0.02,0.04)	(1.00,0.60)	(1.00,0.50)	1
100×100	0.1091	0.8924	0.9567	0.1484	0.8663	(0.02,0.10)	(1.00,0.60)	(1.00,0.50)	2,3
200×200	0.2791	0.8710	0.9323	0.3871	0.8243	(0.10,0.30)	(1.00,0.60)	(1.00,0.50)	2,4,6,9
300×300	0.4225	0.7644	0.8528	0.6295	0.8122	(0.04,0.50)	(1.00,0.60)	(1.00,0.50)	6,12,18,24

**Table 2:** Parameter selection.

Parameter	2Obj	3Obj	Range
**NSGA-II**
$ps_{n}$	**100**	**50**	[25,50,…,300]
$cro_{n}$	**0.50**	**0.80**	[0.05,0.1,0.15,…,0.95]
$mut_{n}$	**0.50**	**0.80**	[0.05,0.1,0.15,…,0.95]
**SPEA2**
$ps_{s}$	**100**	**50**	[25,50,…,300]
${\bar{ps}}_{s}$	**100**	**50**	-
$cro_{s}$	**0.50**	**0.60**	[0.05,0.1,0.15,…,0.95]
$mut_{s}$	**0.50**	**0.70**	[0.05,0.1,0.15,…,0.95]
**MO-VNS**
$neigh_{v}$	**7**	**9**	[4,5,6,…,14]
$ns_{v}$	**2**	**2**	[1,2,3,4,5]
MO-VNS*
$neigh_{v}$	**11**	**10**	[4,5,6,…,14]
$ns_{v}$	**3**	**2**	[1,2,3,4,5]
$per_{v}$	**0.10**	**0.10**	[0.05,0.1,0.15,…,0.95]
**MO-ABC**
$ps_{a}$	**100**	**50**	[25,50,…,300]
$Se_{a}$	**0.50**	**0.25**	[0.30,0.35,0.40,…,0.70]
$limit_{a}$	**30**	**15**	[10,15,20,…,60]
**MO-FA**
$ps_{f}$	**100**	**100**	[25,50,…,300]
$r_{f}$	**0.50**	**0.85**	[0.05,0.1,0.15,…,0.95]
$\beta_{0_{f}}$	**0.75**	**0.70**	[0.05,0.1,0.15,…,0.95]
$\gamma_{f}$	**0.05**	**0.60**	[0.05,0.1,0.15,…,0.95]
$mut_{f}$	**0.60**	**0.10**	[0.05,0.1,0.15,…,0.95]
$when\_sc_{f}$	**0.30**	**0.25**	[0.05,0.1,0.15,…,0.95]
**MO-GSA**
$ps_{ga}$	**100**	**25**	[25,50,…,300]
mutga	**0.40**	**0.20**	[0.05,0.1,0.15,…,0.95]
$when\_sc_{ga}$	**0.05**	**0.05**	[0.05,0.1,0.15,…,0.95]
**MOEA/D**
$CHIMinc_{m}$	**1.30**	**1.30**	[1.00,1.05,…,2.00]
$crow_{m}$	**0.015**	**0.015**	[0.010,0.015,…,0.050]
$neigh_{m}$	**0.55**	**0.05**	[0.05,0.10,…,0.95]
$cro_{m}$	**0.15**	**0.15**	[0.05,0.10,…,0.95]
$mut_{m}$	**0.25**	**0.25**	[0.05,0.10,…,0.95]

**Table 3:** Median hypervolume obtained solving the bi-objective RNPP.

	$\mathrm{MO}-\mathrm{VNS}*(\bar{\mathrm{Hyp}})$					**$\mathrm{MO}-\mathrm{ABC}(\bar{\mathrm{Hyp}})$**				
**$d_{x}\times d_{y}\_r_{c}\left({\tilde{s}}_{r}\right)$**	**Evaluations (Stop Condition)**					**Evaluations (Stop Condition)**				
	**50,000**	**100,000**	**200,000**	**300,000**	**400,000**	**50,000**	**100,000**	**200,000**	**300,000**	**400,000**
**50 × 50_30(1)**	66.79%	66.84%	66.88%	66.94%	66.98%	67.07%	67.07%	67.07%	67.07%	67.07%
**50 × 50_60(1)**	66.57%	66.79%	66.92%	66.96%	67.00%	67.07%	67.07%	67.07%	67.07%	67.07%
**100 × 100_30(2)**	41.90%	42.73%	43.49%	44.69%	44.69%	44.64%	44.64%	44.65%	44.66%	44.66%
**100 × 100_30(3)**	58.17%	58.48%	58.53%	58.52%	58.57%	58.79%	59.11%	59.15%	59.16%	59.18%
**100 × 100_60(2)**	34.46%	34.56%	34.56%	34.58%	34.59%	34.39%	34.40%	34.41%	34.41%	34.41%
**100 × 100_60(3)**	60.74%	61.29%	61.62%	62.20%	62.32%	61.57%	61.95%	62.02%	62.04%	62.06%
**200 × 200_30(2)**	35.44%	37.35%	38.35%	38.66%	39.03%	37.98%	37.98%	37.99%	37.99%
**200 × 200_30(4)**	48.64%	50.13%	51.56%	52.88%	53.71%	48.41%	49.89%	52.59%	53.21%	53.28%
**200 × 200_30(6)**	66.48%	67.06%	67.47%	67.70%	67.80%	59.69%	62.60%	65.80%	67.74%	68.27%
**200 × 200_30(9)**	77.99%	78.97%	79.63%	80.06%	80.31%	73.07%	75.35%	77.40%	78.79%	80.08%
**200 × 200_60(2)**	23.43%	24.26%	24.51%	24.59%	24.66%	24.73%	24.82%	24.83%	24.84%	24.85%
**200 × 200_60(4)**	61.83%	61.95%	62.16%	62.27%	62.39%	59.82%	61.88%	62.45%	62.50%	62.55%
**200 × 200_60(6)**	76.83%	77.42%	77.84%	78.06%	78.30%	73.92%	75.29%	77.00%	78.19%	78.70%
**200 × 200_60(9)**	89.74%	90.46%	91.08%	91.37%	91.43%	87.02%	88.66%	90.19%	91.19%	92.09%
**300 × 300_30(6)**	41.09%	41.66%	42.18%	42.42%	42.56%	40.24%	41.45%	42.51%	43.09%	43.46%
**300 × 300_30(12)**	47.31%	47.95%	48.50%	48.77%	48.87%	47.46%	49.40%	50.52%	51.04%	51.38%
**300 × 300_30(18)**	51.31%	52.08%	52.78%	53.28%	53.51%	51.90%	54.31%	56.07%	56.79%	57.41%
**300 × 300_30(24)**	55.94%	57.58%	58.86%	59.26%	59.64%	55.41%	59.05%	61.93%	63.46%	64.24%
**300 × 300_60(6)**	37.45%	37.89%	38.31%	38.57%	38.73%	**34.55%**	**35.89%**	**37.86%**	**39.17%**	**39.94%**
**300 × 300_60(12)**	56.61%	57.35%	57.87%	58.18%	58.32%	**52.61%**	**54.36%**	**55.75%**	**56.63%**	**57.30%**
**300 × 300_60(18)**	63.11%	63.67%	64.08%	64.34%	64.48%	**62.55%**	**63.96%**	**65.31%**	**65.93%**	**66.37%**
**300 × 300_60(24)**	67.86%	68.47%	69.00%	69.25%	69.46%	**67.46%**	**68.78%**	**69.86%**	**70.41%**	**70.78%**
	$\mathrm{MO}-\mathrm{VNS}(\bar{\mathrm{Hyp}})$					$\mathrm{MO}-\mathrm{FA}(\bar{\mathrm{Hyp}})$				
**$d_{x}\times d_{y}\_r_{c}\left({\tilde{s}}_{r}\right)$**	**Evaluations (Stop Condition)**					**Evaluations (Stop Condition)**				
	**50,000**	**100,000**	**200,000**	**300,000**	**400,000**	**50,000**	**100,000**	**200,000**	**300,000**	**400,000**
**50 × 50_30(1)**	63.08%	67.03%	67.03%	67.03%	67.03%	**67.07%**	**67.07%**	**67.07%**	**67.07%**	**67.07%**
**50 × 50_60(1)**	67.03%	67.03%	67.03%	67.03%	67.03%	**67.07%**	**67.07%**	**67.07%**	**67.07%**	**67.07%**
**100 × 100_30(2)**	43.62%	44.67%	44.69%	44.69%	44.69%	**44.66%**	**44.68%**	**44.69%**	**44.69%**	**44.69%**
**100 × 100_30(3)**	58.33%	58.65%	58.85%	59.09%	59.24%	**58.47%**	**58.59%**	**58.74%**	**58.83%**	**58.84%**
**100 × 100_60(2)**	34.55%	34.58%	34.60%	34.61%	34.63%	**33.99%**	**34.10%**	**34.25%**	**34.36%**	**34.41%**
**100 × 100_60(3)**	61.41%	62.05%	62.17%	62.25%	62.33%	**61.54%**	**61.84%**	**62.00%**	**62.08%**	**62.14%**
**200 × 200_30(2)**	37.63%	38.82%	39.93%	40.75%	41.07%	**38.06%**	**38.60%**	**41.02%**	**41.03%**	**41.05%**
**200 × 200_30(4)**	51.99%	53.29%	54.31%	54.74%	54.96%	**49.95%**	**50.63%**	**50.95%**	**51.20%**	**51.16%**
**200 × 200_30(6)**	64.36%	65.47%	66.31%	66.63%	66.87%	**66.70%**	**67.29%**	**67.52%**	**67.71%**	**67.68%**
**200 × 200_30(9)**	74.57%	75.84%	77.12%	77.75%	78.28%	**78.92%**	**80.26%**	**80.76%**	**81.19%**	**81.30%**
**200 × 200_60(2)**	24.43%	24.55%	24.65%	24.68%	24.72%	**24.38%**	**24.52%**	**24.61%**	**24.70%**	**24.74%**
**200 × 200_60(4)**	61.28%	61.68%	61.95%	62.17%	62.29%	**61.61%**	**61.73%**	**61.79%**	**61.83%**	**61.86%**
**200 × 200_60(6)**	75.95%	76.75%	77.22%	77.61%	77.86%	**76.38%**	**76.99%**	**77.19%**	**77.24%**	**77.34%**
**200 × 200_60(9)**	89.42%	90.21%	91.02%	91.39%	91.59%	**90.84%**	**91.30%**	**91.51%**	**91.64%**	**91.71%**
**300 × 300_30(6)**	39.85%	40.57%	41.22%	41.67%	41.89%	**40.68%**	**41.15%**	**41.40%**	**41.58%**	**41.65%**
**300 × 300_30(12)**	45.28%	46.39%	47.45%	47.97%	48.33%	**49.20%**	**50.25%**	**51.06%**	**51.25%**	**51.41%**
**300 × 300_30(18)**	48.49%	49.51%	50.47%	51.04%	51.59%	**54.15%**	**56.56%**	**58.06%**	**58.72%**	**59.13%**
**300 × 300_30(24)**	50.54%	51.88%	52.92%	53.75%	54.28%	**59.68%**	**63.40%**	**65.98%**	**66.58%**	**66.90%**
**300 × 300_60(6)**	35.79%	36.39%	37.26%	37.69%	38.11%	**38.09%**	**38.57%**	**38.83%**	**38.90%**	**38.99%**
**300 × 300_60(12)**	53.68%	54.99%	55.95%	56.51%	56.86%	**58.28%**	**58.97%**	**59.40%**	**59.55%**	**59.64%**
**300 × 300_60(18)**	61.69%	62.75%	63.79%	64.26%	64.57%	**65.19%**	**66.47%**	**66.93%**	**67.21%**	**67.30%**
**300 × 300_60(24)**	66.52%	67.44%	68.28%	68.69%	68.95%	**69.25%**	**70.59%**	**71.34%**	**71.59%**	**71.69%**
	$\mathrm{MO}-\mathrm{GSA}(\bar{\mathrm{Hyp}})$					$\mathrm{MOEA}/D(\bar{\mathrm{Hyp}})$				
**$d_{x}\times d_{y}\_r_{c}\left({\tilde{s}}_{r}\right)$**	**Evaluations (Stop Condition)**					**Evaluations (Stop Condition)**				
	**50,000**	**100,000**	**200,000**	**300,000**	**400,000**	**50,000**	**100,000**	**200,000**	**300,000**	**400,000**
**50 × 50_30(1)**	**67.07%**	**67.07%**	**67.07%**	**67.07%**	**67.07%**	**67.07%**	**67.07%**	**67.07%**	**67.07%**	**67.07%**
**50 × 50_60(1)**	**67.07%**	**67.07%**	**67.07%**	**67.07%**	**67.07%**	**67.07%**	**67.07%**	**67.07%**	**67.07%**	**67.07%**
**100 × 100_30(2)**	43.51%	44.10%	44.46%	44.53%	44.64%	**44.22%**	**44.25%**	**44.32%**	**44.34%**	**44.35%**
**100 × 100_30(3)**	55.57%	56.21%	57.32%	58.03%	58.24%	**58.30%**	**58.39%**	**58.41%**	**58.43%**	**58.43%**
**100 × 100_60(2)**	33.59%	33.85%	34.20%	34.33%	34.39%	**33.33%**	**33.61%**	**33.76%**	**33.92%**	**34.04%**
**100 × 100_60(3)**	60.47%	61.07%	61.61%	61.81%	61.89%	**57.79%**	**58.09%**	**58.40%**	**58.65%**	**58.81%**
**200 × 200_30(2)**	37.36%	37.46%	37.92%	38.51%	38.87%	**37.25%**	**37.57%**	**37.86%**	**37.97%**	**38.25%**
**200 × 200_30(4)**	47.42%	48.89%	51.24%	52.56%	53.02%	**48.08%**	**48.71%**	**49.68%**	**50.01%**	**50.63%**
**200 × 200_30(6)**	60.76%	63.50%	65.13%	65.90%	66.44%	**60.91%**	**63.55%**	**63.35%**	**63.57%**	**63.98%**
**200 × 200_30(9)**	72.35%	74.83%	76.78%	77.69%	78.48%	**74.45%**	**75.41%**	**76.25%**	**76.56%**	**76.78%**
**200 × 200_60(2)**	22.69%	23.38%	24.28%	24.37%	24.57%	**23.82%**	**23.98%**	**24.11%**	**24.17%**	**24.18%**
**200 × 200_60(4)**	58.79%	60.20%	61.16%	61.41%	61.66%	**57.01%**	**57.68%**	**58.15%**	**58.32%**	**58.42%**
**200 × 200_60(6)**	72.31%	74.04%	75.77%	76.58%	77.02%	**70.97%**	**71.72%**	**72.19%**	**72.50%**	**72.83%**
**200 × 200_60(9)**	83.86%	86.74%	89.78%	90.54%	91.09%	**84.15%**	**84.71%**	**85.28%**	**85.58%**	**85.74%**
**300 × 300_30(6)**	**37.70%**	**38.89%**	**39.95%**	**40.52%**	**40.89%**	**37.78%**	**38.41%**	**38.95%**	**39.21%**	**39.41%**
**300 × 300_30(12)**	**43.71%**	**45.79%**	**47.68%**	**48.44%**	**49.04%**	**46.02%**	**46.68%**	**47.38%**	**47.84%**	**48.04%**
**300 × 300_30(18)**	**48.67%**	**51.12%**	**54.09%**	**55.25%**	**56.38%**	**53.29%**	**54.00%**	**54.65%**	**55.09%**	**55.30%**
**300 × 300_30(24)**	**56.82%**	**60.25%**	**62.50%**	**63.56%**	**64.47%**	**58.03%**	**59.22%**	**60.05%**	**60.44%**	**60.70%**
**300 × 300_60(6)**	**33.80%**	**35.10%**	**36.81%**	**37.57%**	**37.99%**	**36.51%**	**36.86%**	**37.40%**	**37.56%**	**37.65%**
**300 × 300_60(12)**	**54.37%**	**56.14%**	**57.33%**	**57.97%**	**58.54%**	**55.13%**	**56.01%**	**56.61%**	**56.80%**	**56.96%**
**300 × 300_60(18)**	**62.37%**	**63.81%**	**65.78%**	**66.70%**	**67.36%**	**62.97%**	**63.60%**	**64.27%**	**64.56%**	**64.77%**
**300 × 300_60(24)**	**66.15%**	**67.92%**	**70.01%**	**71.17%**	**71.72%**	**67.65%**	**68.27%**	**68.79%**	**69.11%**	**69.31%**

**Table 4:** Based on hypervolume, percentage of average test cases where a metaheuristic is significant better than any other solving the bi-objective RNPP.

	Instance Size				
	50×50	100×100	200×200	300×300	All
**NSGA-II**	0.00%	2.35%	2.44%	2.27%	2.17%
**SPEA2**	0.00%	0.29%	1.11%	5.72%	2.52%
**MO-VNS**	6.31%	11.37%	10.03%	2.11%	7.16%
MO-VNS*	4.85%	8.53%	10.03%	7.45%	8.40%
**MO-ABC**	9.71%	9.41%	8.55%	8.75%	8.88%
**MO-FA**	9.71%	9.51%	10.77%	13.71%	11.49%
**MO-GSA**	9.71%	5.00%	4.72%	5.83%	5.57%
**MOEA/D**	9.71%	3.53%	2.34%	4.16%	3.81%

**Table 5:** Average set coverage for a metaheuristic compared to any other solving the bi-objective RNPP.

	Instance Size				
	50×50	100×100	200×200	300×300	All
**NSGA-II**	26.06%	14.94%	19.95%	30.79%	23.54%
**SPEA2**	30.63%	10.05%	14.90%	45.46%	26.56%
**MO-VNS**	88.28%	87.56%	66.62%	20.83%	53.12%
MO-VNS*	44.63%	83.11%	68.92%	51.86%	63.82%
**MO-ABC**	100.00%	75.90%	54.78%	44.29%	58.92%
**MO-FA**	100.00%	75.93%	77.52%	86.47%	81.47%
**MO-GSA**	100.00%	45.45%	36.58%	38.87%	41.94%
**MOEA/D**	90.00%	34.30%	18.38%	25.59%	27.21%

**Table 6:** Median hypervolume obtained solving the three-objective RNPP.

	**$\mathrm{MO}-\mathrm{VNS}(\bar{\mathrm{Hyp}})$**					**$\mathrm{MO}-\mathrm{FA}(\bar{\mathrm{Hyp}})$**				
**$d_{x}\times d_{y}\_r_{c}\left({\tilde{s}}_{r}\right)$**	**Evaluations (Stop condition)**					**Evaluations (Stop condition)**				
	**50,000**	**100,000**	**200,000**	**300,000**	**400,000**	**50,000**	**100,000**	**200,000**	**300,000**	**400,000**
**50 × 50_30(1)**	**64.58%**	**64.58%**	**64.59%**	**64.59%**	**64.60%**	64.63%	64.63%	64.63%	64.63%	64.63%
**100 × 100_30(2)**	**41.73%**	**41.77%**	**41.81%**	**41.81%**	**41.82%**	41.66%	41.71%	41.75%	41.77%	41.78%
**100 × 100_30(3)**	**54.87%**	**55.19%**	**55.45%**	**55.55%**	**55.59%**	54.79%	55.19%	55.29%	55.35%	55.38%
**200 × 200_30(2)**	**31.98%**	**33.18%**	**34.41%**	**35.37%**	**35.84%**	35.05%	35.57%	35.98%	36.00%	36.06%
**200 × 200_30(4)**	**41.94%**	**43.76%**	**45.09%**	**45.60%**	**46.05%**	43.65%	44.58%	45.21%	45.56%	45.91%
**200 × 200_30(6)**	**52.49%**	**54.94%**	**57.05%**	**58.10%**	**58.74%**	55.22%	56.54%	57.89%	58.38%	58.96%
**200 × 200_30(9)**	**63.30%**	**65.41%**	**67.30%**	**68.39%**	**69.13%**	65.87%	67.83%	69.82%	70.50%	70.94%
**300 × 300_30(6)**	**30.37%**	**30.97%**	**31.61%**	**31.97%**	**32.18%**	30.28%	31.39%	32.59%	32.90%	33.02%
**300 × 300_30(12)**	**34.06%**	**35.13%**	**36.08%**	**36.75%**	**37.13%**	34.63%	36.24%	37.76%	38.25%	39.17%
**300 × 300_30(18)**	**36.62%**	**37.80%**	**38.95%**	**39.58%**	**40.09%**	37.97%	39.63%	41.18%	41.90%	42.54%
**300 × 300_30(24)**	**39.59%**	**40.96%**	**42.20%**	**42.90%**	**43.45%**	40.83%	42.88%	44.35%	45.13%	45.59%
	$\mathrm{MO}-\mathrm{GSA}(\bar{\mathrm{Hyp}})$					$\mathrm{MOEA}/D(\bar{\mathrm{Hyp}})$				
**$d_{x}\times d_{y}\_r_{c}\left({\tilde{s}}_{r}\right)$**	**Evaluations (Stop condition)**					**Evaluations (Stop condition)**				
	**50,000**	**100,000**	**200,000**	**300,000**	**400,000**	**50,000**	**100,000**	**200,000**	**300,000**	**400,000**
**50 × 50_30(1)**	**64.56%**	**64.56%**	**64.56%**	**64.56%**	**64.56%**	64.62%	64.62%	64.63%	64.63%	64.63%
**100 × 100_30(2)**	**39.70%**	**40.24%**	**40.77%**	**41.08%**	**41.21%**	41.07%	41.20%	41.31%	41.35%	41.39%
**100 × 100_30(3)**	**52.40%**	**53.15%**	**53.75%**	**54.05%**	**54.18%**	54.82%	55.12%	55.36%	55.42%	55.48%
**200 × 200_30(2)**	**32.53%**	**32.82%**	**33.21%**	**33.50%**	**33.59%**	32.32%	32.76%	33.27%	33.54%	33.71%
**200 × 200_30(4)**	**41.03%**	**42.97%**	**44.66%**	**45.62%**	**45.96%**	43.85%	44.82%	46.18%	46.72%	46.69%
**200 × 200_30(6)**	**50.75%**	**52.58%**	**55.82%**	**57.24%**	**57.98%**	57.48%	58.62%	59.56%	60.01%	60.38%
**200 × 200_30(9)**	**61.53%**	**63.79%**	**67.15%**	**69.10%**	**69.64%**	69.60%	70.96%	72.16%	72.87%	73.35%
**300 × 300_30(6)**	**29.05%**	**29.82%**	**30.81%**	**31.27%**	**31.47%**	30.54%	31.25%	31.82%	32.08%	32.26%
**300 × 300_30(12)**	**33.49%**	**34.74%**	**36.40%**	**37.68%**	**38.00%**	36.39%	37.56%	38.48%	38.85%	39.11%
**300 × 300_30(18)**	**37.57%**	**38.85%**	**41.62%**	**43.18%**	**43.72%**	40.53%	42.22%	43.92%	44.74%	45.21%
**300 × 300_30(24)**	**42.37%**	**44.51%**	**47.76%**	**49.86%**	**50.33%**	45.09%	47.51%	49.82%	51.04%	51.75%
	$\mathrm{MO}-\mathrm{VNS}*(\bar{\mathrm{Hyp}})$				
**$d_{x}\times d_{y}\_r_{c}\left({\tilde{s}}_{r}\right)$**	**Evaluations (Stop condition)**									
	**50,000**	**100,000**	**200,000**	**300,000**	**400,000**					
**50 × 50_30(1)**	64.60%	64.61%	64.62%	64.62%	64.63%					
**100 × 100_30(2)**	41.75%	41.79%	41.81%	41.81%	41.82%					
**100 × 100_30(3)**	54.96%	55.17%	55.45%	55.56%	55.61%					
**200 × 200_30(2)**	31.76%	34.00%	34.60%	35.22%	35.49%					
**200 × 200_30(4)**	42.81%	44.38%	45.24%	45.78%	46.14%					
**200 × 200_30(6)**	54.27%	56.20%	56.80%	57.13%	57.47%					
**200 × 200_30(9)**	63.48%	64.30%	65.33%	65.87%	66.45%					
**300 × 300_30(6)**	30.39%	30.93%	31.23%	31.34%	31.40%					
**300 × 300_30(12)**	33.88%	34.56%	35.31%	35.68%	35.83%					
**300 × 300_30(18)**	37.04%	37.83%	38.48%	38.77%	39.01%					
**300 × 300_30(24)**	40.14%	40.85%	41.48%	41.79%	41.95%					

**Table 7:** Based on hypervolume, percentage of average test cases where a metaheuristic is significant better than any other solving the three-objective RNPP.

	Instance Size				
	50×50	100×100	200×200	300×300	All
**NSGA-II**	0.00%	3.15%	5.13%	1.15%	2.68%
**SPEA2**	0.00%	3.78%	7.00%	5.01%	4.89%
**MO-VNS**	5.73%	11.55%	6.13%	4.18%	6.37%
MO-VNS*	7.63%	10.92%	5.50%	1.98%	5.41%
**MO-ABC**	9.54%	7.35%	0.00%	8.46%	5.65%
**MO-FA**	13.36%	8.61%	12.50%	11.90%	11.62%
**MO-GSA**	3.82%	0.00%	2.75%	6.16%	3.65%
**MOEA/D**	9.92%	4.62%	11.00%	11.17%	9.74%

**Table 8:** Average set coverage for a metaheuristic compared to any other solving the three-objective RNPP.

	Instance size				
	50×50	100×100	200×200	300×300	All
**NSGA-II**	21.00%	23.95%	39.52%	22.80%	28.92%
**SPEA2**	21.24%	28.12%	40.54%	37.38%	35.38%
**MO-VNS**	55.83%	72.36%	48.77%	40.80%	50.80%
MO-VNS*	67.86%	73.19%	49.37%	33.51%	49.61%
**MO-ABC**	40.48%	43.20%	28.75%	63.12%	44.94%
**MO-FA**	72.00%	65.68%	83.71%	51.12%	67.52%
**MO-GSA**	81.78%	12.74%	10.68%	30.43%	24.70%
**MOEA/D**	38.69%	12.15%	20.43%	18.66%	19.94%

## Abbreviations

ABC	Artificial Bee Colony
AEC	Average Energy Cost
ASA	Average Sensitivity Area
CHIM	Convex Hull of Individual Minima
C-RNPP	Constrained Relay Node Placement Problem
EA	Evolutionary Algorithm
FA	Firefly Algorithm
GA	Genetic Algorithm
GSA	Gravitational Search Algorithm
MO	Multi-Objective
MO-ABC	Multi-Objective Artificial Bee Colony
MOEA/D	Multi-Objective Evolutionary Algorithm Based on Decomposition
MO-FA	Multi-Objective Firefly Algorithm
MO-GSA	Multi-Objective Gravitational Search Algorithm
MO-VNS	Multi-Objective Variable Neighborhood Search
NBI	Normal Boundary Intersection
NP	Non-deterministic Polynomial-time
NR	Network Reliability
NSGA-II	Non-dominated Sorting Genetic Algorithm-II
PSO	Particle Swarm Optimization
QoS	Quality of Service
RN	Relay Node
RNPP	Relay Node Placement Problem
SIA	Swarm Intelligence Algorithm
SPEA2	Strength Pareto Evolutionary Algorithm 2
TA	Trajectory Algorithm
T-WSN	Traditional Wireless Sensor Network
ST	Single-Tiered
ST-WSN	Single-Tiered Wireless Sensor Network
TT-WSN	Two-Tiered Wireless Sensor Network
WSN	Wireless Sensor Network
α	path loss exponent, α∈[2,4].
A(t)	sensitivity area provided by the WSN at time t>0∈τ.
$a_{p}\left(t\right)$	variable assuming 1 if there is at least one sensor $i\in S_{s}\left(t\right)$ at a distance to the demand point $p\in {\tilde{D}}_{p}\left(t\right)$ lower than $r_{s}$.
amp	energy cost per bit of the power amplifier, amp>0.
β	transmission quality parameter, β>0.
$\beta_{0_{f}}$	parameter determining the performance of the movement operator based on attractiveness in MO-FA, β∈[0,1].
*c*	sink coordinates, c=(x,y) where $x\in [0,d_{x}]$ and $y\in [0,d_{y}]$.
$CHIMinc_{m}$	parameter defining how the extreme points of the CHIM are reassigned to increase the search area in MOEA/D, $CHIMinc_{m}\in \left[1,\infty \right)$.
$co_{th}$	coverage threshold, $co_{th}\in \left[0,1\right]$.
$cro_{m}$	crossover probability in the MOEA/D algorithm.
$cro_{n}$	crossover probability in the NSGA-II algorithm.
$cro_{s}$	crossover probability in the SPEA2 algorithm.
$crow_{m}$	distance between two consecutive reference points in MOEA/D, $crow_{m}\in \left(0,\infty \right)$.
${\tilde{D}}_{p}\left(t\right)$	set of demand points at time t>0, $\forall p\in {\tilde{D}}_{p},\;p=\left(x,y\right)$ where $x\in [0,d_{x}]$ and $y\in [0,d_{y}]$.
${\tilde{d}}_{p}\left(t\right)$	number of demand points. It is the cardinal of ${\tilde{D}}_{p}\left(t\right)$.
$d_{pn}$	distance between two neighbouring demand points.
$djp_{i}^{c}$	number of disjoint paths between the sensor $i\in {\tilde{S}}_{s}$ and the sink node.
$d_{x}$	width of the surface, $d_{x}>0$.
$d_{y}$	height of the surface, $d_{y}>0$.
$E_{1}$, $E_{2}$	extreme points depending on $F_{1}$ and $F_{2}$; $E_{1},E_{2}\in Y$.
$Ec_{i}\left(t\right)$	energy charge of a sensor $i\in S_{s}\left(t\right)$ at time *t*.
$Ee_{i}\left(t\right)$	energy expenditure of a sensor $i\in S_{s}\left(t\right)$ at time t>0.
err	local channel error, err∈[0,1].
$F_{1}$	extreme point $(maxF\left(f_{1}\right),minF\left(f_{2}\right)$ delimiting the objective space, $F_{1}\in Y$.
$f_{1}$	AEC of the sensors over the network lifetime.
$F_{2}$	extreme point $(minF\left(f_{1}\right),maxF\left(f_{2}\right)$ delimiting the objective space, $F_{2}\in Y$.
$f_{2}$	ASA provided by the WSN over the network lifetime.
$f_{3}$	NR provided by the WSN at the beginning of the network lifetime.
$F_{m}^{g}$	auxiliary population of undefined size in MOEA/D, saving the non-dominated solutions found until the *g*-iteration.
$g(x:r^{m},\vec{v_{n}})$	function of the *m*-th single -objective minimization problem in MOEA/D.
$h_{l}^{i,c}$	number of hops in the *l*-th disjoint path between $i\in {\tilde{S}}_{s}$ and the sink node.
iec	initial energy charge of the sensors, iec>0.
*k*	information packet size in bits, k>0.
$k_{a}$	random value representing the number of nearest solution used to obtain a new solution
	by an scout bee in the MO-ABC algorithm, $k_{a}=2,\ldots ,11$.
$\lambda_{f}$	parameter determining the performance of the movement operator based on attractiveness in MO-FA, λ∈(0,∞).
$limit_{a}$	threshold determining when a solution managed by an employed bee should be managed by an scout bee in MO-ABC.
maxF(·)	lower bounds of a fitness function.
minF(·)	upper bounds of a fitness function.
$mut_{f}$	mutation probability in the MO-FA algorithm.
$mut_{ga}$	mutation probability in the MO-GSA algorithm.
$mut_{m}$	mutation probability in the MOEA/D algorithm.
$mut_{n}$	mutation probability in the NSGA-II algorithm.
$mut_{s}$	mutation probability in the SPEA2 algorithm.
$mut_{v}$	mutation probability in the MO-VSN* algorithm.
$n_{1}$, $n_{2}$	coordinates of the normal vector $\vec{v_{n}}$ to the plane I.
$n_{E_{1},E_{2}}$	number of divisions on the segment $\bar{E_{1}E_{2}}$.
$neigh_{m}$	number of neighbouring reference points associated to a reference point in MOEA/D.
$neigh_{v}$	number of neighbourhood structures in the MO-VSN algorithm.
$ns_{v}$	parameter limiting how different could be a solution according to the neighbourhood structure selected in MO-VNS, $ns_{v}\in \left[1,\infty \right)$.
$P_{i}\left(t\right)$	number of packets sent by the sensor $i\in S_{s}\left(t\right)$ at time t>0.
$P_{a}^{g}$	population of size $ps_{a}$ in the MO-ABC algorithm.
$P_{f}^{g}$	population of size $ps_{f}$ saving the the fireflies at the beginning of the iteration *g* in MO-FA.
$P_{ga}^{g}$	population of size $ps_{g}$ saving the the objects at the beginning of *g*, before acting gravitational forces in the MO-GSA algorithm.
$P_{m}^{g}$	regular population in MOEA/D, where each individual is associated to a reference point.
$P_{n}^{g}$	population of size $ps_{n}$ saving the parents of the iteration *g* in the NSGA-II algorithm.
$P_{s}^{g}$	regular population of size $ps_{s}$ in the SPEA2 algorithm.
${\bar{P}}_{s}^{g}$	auxiliary population of size ${\bar{ps}}_{s}$ in the SPEA2 algorithm.
$P_{v}^{g}$	population of unlimited size, saving only non-dominated solutions during the iteration *g* in the MO-VNS algorithm.
$ps_{a}$	population size in the MO-ABC algorithm. It is the cardinal of $P_{a}^{g}$.
$ps_{m}$	number of points evenly distributed in the plane I. It is the cardinal of *Y*.
$ps_{f}$	population size in the MO-FA algorithm. It is the cardinal of $P_{f}^{g}$ and $Q_{f}^{g}$.
$ps_{ga}$	population size in the MO-GSA algorithm. It is the cardinal of $P_{ga}^{g}$ and $Q_{ga}^{g}$.
$ps_{n}$	population size in the NSGA-II algorithm. It is the cardinal of $P_{n}^{g}$ and $Q_{n}^{g}$.
$ps_{s}$	regular population size in the SPEA2 algorithm. It is the cardinal of $P_{s}^{g}$.
${\bar{ps}}_{s}$	auxiliary population size in the SPEA2 algorithm. It is the cardinal of ${\bar{P}}_{s}^{g}$.
$Q_{f}^{g}$	population of size $ps_{f}$ saving the resulting fireflies after applying the attractiveness mechanism in $P_{f}^{g}$ in the MO-FA algorithm.
$Q_{ga}^{g}$	population of size $ps_{g}$ saving the resulting objects after applying the forces in $P_{ga}^{g}$ in the MO-GSA algorithm.
$Q_{n}^{g}$	population of size $ps_{n}$ saving the offspring generated based on $P_{n}^{g}$ in the NSGA-II algorithm.
$re_{i}$	reliability of the sensor $i\in {\tilde{S}}_{s}$.
$r_{c}$	communication radius, $r_{c}>0$.
$Rp_{i}\left(t\right)$	number of relayed packets sent by the sensor $i\in S_{s}\left(t\right)$ at time t>0.
$r_{f}$	parameter determining the performance of the movement operator based on attractiveness in MO-FA, r∈[0,1].
$r^{m}$	*m*-th point of *Y*.
$r_{1}^{m}$, $r_{2}^{m}$	coordinates of the vector $r^{m}$.
$r_{s}$	sensitivity radius, $r_{s}>0$.
$se_{a}$	percentage of solutions in $P_{a}^{g}$ managed by employed forager bees in MO-ABC.
${\tilde{S}}_{r}$	set of RN coordinates, $\forall r\in {\tilde{S}}_{r}$, r=(x,y) where $x\in [0,d_{x}]$ and $y\in [0,d_{y}]$.
${\tilde{s}}_{r}$	number of RNs. It is the cardinal of ${\tilde{S}}_{r}$.
${\tilde{S}}_{s}$	set of initial sensor coordinates, $\forall i\in {\tilde{S}}_{s}$, i=(x,y), where $x\in [0,d_{x}]$ and $y\in [0,d_{y}]$.
${\tilde{s}}_{s}$	number of initial sensors. It is the cardinal of ${\tilde{S}}_{s}$.
$S_{s}\left(t\right)$	set of sensor coordinates, holding that the energy charge is greater than 0 and that there is any path to the sink node, both at time t>0, $S_{s}\left(t\right)\subseteq {\tilde{S}}_{s}$, $\forall i\in S_{s}$, i=(x,y), where $x\in [0,d_{x}]$ and $y\in [0,d_{y}]$.
$s_{s}\left(t\right)$	number of sensors, holding that the energy charge is greater than 0 and that there is any path to the sink node, both at time t>0. It is the cardinal of $S_{s}\left(t\right)$, $s_{s}\left(t\right)\leq {\tilde{s}}_{s}$.
$S_{v}^{g}$	population of unlimited size saving the solutions from $P_{v}^{g}$ considered to explore the search space during the iteration *g* in the MO-VNS algorithm.
τ	set of time periods, τ={0,1,2,…}.
*t*	time instant, t>0.
$t_{n}$	network lifetime of the WSN based on the coverage threshold $co_{th}$.
$\vec{v_{n}}$	normal vector $\left(n_{1},n_{2}\right)$ to the plane I.
$when\_sc_{f}$	threshold defining when the anti-stagnation mechanism is performed in MO-FA, $when\_sc_{f}\in \left[0,1\right]$.
$when\_sc_{ga}$	threshold defining when the anti-stagnation mechanism is performed in MO-GSA, $when\_sc_{ga}\in \left[0,1\right]$.
$w_{i}^{c}\left(t\right)$	variable which provides the next device in the minimum path between $i\in S_{s}\left(t\right)$ and the sink node at t>0, $w_{i}^{c}\left(t\right)\in \left\{S_{s}\left(t\right)\cup {\tilde{S}}_{r}\right\}+c-i$.
*Y*	number of points evenly distributed in the plane I, $Y=\{r^{1},\ldots ,r^{ps_{m}}\}$.
$z_{j,i}^{c}\left(t\right)$	variable assuming 1 if $i\in S_{s}\left(t\right)$ is in the minimum path between $j\in S_{s}\left(t\right)$ and the sink node *c* at t>0, and 0 otherwise.
